# Body composition-defined sarcopenia phenotypes, diabetes complications, and CGM-derived glycemic profiles in frail older adults with diabetes: a cross-sectional study

**DOI:** 10.1016/j.jnha.2026.100947

**Published:** 2026-08-08

**Authors:** Ernesto Guevara, Andreu Simó-Servat, Verónica Perea, Carmen Quirós, Carlos Puig-Jové, Francesc Formiga, María-José Barahona

**Affiliations:** a Department of Geriatrics, Hospital Universitari Mútua Terrassa, Terrassa, Barcelona, Spain; b University of Barcelona, Barcelona, Spain; c Department of Endocrinology, Hospital Universitari Mútua Terrassa, Terrassa, Barcelona, Spain; d Department of Internal Medicine, Instituto de Investigación Biomédica de Bellvitge (IDIBELL), Hospital Universitari de Bellvitge, Bellvitge, Barcelona, Spain

**Keywords:** Body composition, Continuous glucose monitoring, Diabetes mellitus, Frailty, Sarcopenia

## Abstract

**Background and aims:**

Sarcopenia in older adults with diabetes may encompass clinically distinct phenotypes. We examined whether body composition-defined phenotypes differed in diabetes complications, insulin resistance-related metabolic features, and continuous glucose monitoring (CGM)-derived glycemic profiles.

**Methods:**

This cross-sectional study included 109 adults aged 70 years or older with diabetes and frailty. Sarcopenia was defined according to the European Working Group on Sarcopenia in Older People 2 criteria; adiposity was defined using body mass index, body fat percentage, and central obesity. Analyses were mainly descriptive; exploratory logistic regression assessed poor CGM time in range (TIR), defined as TIR below 70%.

**Results:**

Sarcopenia was identified in 86 participants (78.9%). After exclusion of 2 participants in a small non-sarcopenic with adiposity subgroup, 107 participants formed three phenotypes. Sarcopenic obesity showed an insulin resistance-related cluster comprising greater central adiposity, higher triglycerides and TyG index, metabolic syndrome in all participants, and greater insulin requirements. Sarcopenia without adiposity showed more neuropathy, albuminuria, cerebrovascular disease, and lower TIR despite similar glycated hemoglobin. Glycated hemoglobin was the only independent correlate of poor TIR; phenotype was not significant overall.

**Conclusions:**

Phenotypes showed distinct clinical, insulin resistance-related, and CGM profiles. These preliminary findings support phenotype-aware assessment but require confirmation in larger longitudinal studies before informing management.

## 1 Introduction

Sarcopenia is increasingly recognized as a clinically relevant condition in older adults with diabetes. Beyond its effects on strength and physical performance, sarcopenia may reflect broader metabolic vulnerability because skeletal muscle is central to glucose disposal and insulin sensitivity. Contemporary consensus documents support its clinical relevance while acknowledging continuing challenges in definition and phenotypic characterization [1,2].

In older adults with diabetes, sarcopenia often coexists with frailty, multimorbidity, and long disease duration [3,4]. Frailty detection in diabetes is clinically relevant, and meta-analytic evidence links frailty with adverse outcomes in this population [5,6]. However, sarcopenia in this setting is unlikely to be metabolically uniform.

Sarcopenic obesity has emerged as a clinically important phenotype in which excess adiposity coexists with impaired muscle health and may amplify insulin resistance-related abnormalities [7,8]. However, not all sarcopenic individuals have excess adiposity, and the metabolic implications of sarcopenia without adiposity in older adults with diabetes remain insufficiently characterized. Epidemiologic syntheses further indicate that sarcopenic obesity is common in older adults and associated with frailty and adverse outcomes [9,10].

Insulin resistance is central to sarcopenic obesity because excess adiposity and reduced muscle mass may jointly impair glucose disposal and promote metabolic dysfunction [7,8]. Glycemic characterization in diabetes has also moved beyond glycated hemoglobin (HbA1c) alone. CGM provides information on time in range and glycemic variability, which may be particularly relevant in clinically complex older adults [11,12]. The triglyceride-glucose (TyG) index adds pragmatic information on an insulin resistance-related metabolic pattern, although its interpretation remains context-dependent in established diabetes [13,14].

We aimed to determine the prevalence of sarcopenia and to describe whether body composition-defined sarcopenia phenotypes differed in diabetes-related complications, metabolic characteristics, and CGM-derived glycemic profiles among frail older adults with diabetes.

## 2 Subjects, materials and methods

This cross-sectional study used a prospectively maintained institutional database from a hospital-based geriatric-endocrinology outpatient program in Barcelona, Spain. Eligible participants were community-dwelling adults aged 70 years or older with diabetes mellitus and established frailty. Exclusion criteria were advanced cognitive impairment precluding valid assessment and medical contraindications to functional testing.

Frailty was defined using a combined pragmatic approach based on instruments routinely applied in the program. Participants were considered frail when they met criteria on at least one physical frailty instrument (the Physical Frailty Phenotype [15] or FRAIL scale [16]) and at least one multidimensional or global frailty instrument (the 36-item Frailty Index [17] or Clinical Frailty Scale [18]).

Sarcopenia was diagnosed according to the European Working Group on Sarcopenia in Older People 2 algorithm, using low muscle strength as the primary criterion and low muscle quantity for confirmation [1]. Handgrip strength was measured with a calibrated Jamar hydraulic hand dynamometer (Model 5030J1, Performance Health, Warrenville, IL, USA), with participants seated and the elbow flexed at 90 ° alongside the trunk; the highest value from three trials per hand was retained. Skeletal muscle mass adjusted by body weight was assessed by multifrequency bioelectrical impedance analysis (Tanita MC-780MA); low muscle quantity was defined using prespecified sex-specific skeletal muscle mass/weight cutoffs of less than 37% in men and less than 27.6% in women. Bioelectrical impedance analysis was used as a pragmatic bedside body composition method, informed by prior work in adults with diabetes [19].

Adiposity was defined using a composite operational approach informed by ESPEN/EASO concepts, integrating body mass index (BMI), body fat percentage, and central obesity measures [7]. Participants were categorized into four predefined phenotypes according to sarcopenia and adiposity status. Because only 2 participants were classified as non-sarcopenic with adiposity, that subgroup was excluded from phenotype comparisons.

HbA1c was measured using standardized laboratory methods. CGM was performed using FreeStyle Libre 2 over 14 days. Metrics included time in range (70−180 mg/dL), time above range, time below range, glucose management indicator, and coefficient of variation; interpretation considered consensus targets for high-risk adults [12]. Metabolic syndrome was defined according to International Diabetes Federation criteria, and the TyG index was calculated as ln[fasting triglycerides × fasting glucose / 2] [20,21].

Continuous variables are presented as mean (standard deviation) or median (interquartile range), and categorical variables as number (percentage). Between-group comparisons used analysis of variance, Kruskal-Wallis, chi-square, or Fisher exact tests, as appropriate. An exploratory multivariable logistic regression model assessed factors associated with poor CGM time-in-range control, defined as time in range below 70%, adjusting for sex, age, diabetes duration, total daily insulin dose, HbA1c, diabetes type, and sarcopenia/adiposity phenotype. Analyses were performed using SPSS version 29.0. The study was approved by the local ethics committee, and all participants provided written informed consent.

## 3 Results

The study included 109 frail older adults with diabetes. Median age was 78.3 years (IQR, 75.2−83.3), 60.6% were women, and median diabetes duration was 27.6 years (IQR, 17.3−35.7). Type 2 diabetes predominated (81.6%). Sarcopenia was identified in 86 participants (78.9%) (Table 1 ).

**Table 1:** Baseline clinical, diabetes-related, and cardiometabolic characteristics according to sarcopenia status.

Variable	Total (n = 109)	Non-sarcopenic (n = 23)	Sarcopenic (n = 86)	P
Age, median (IQR), years	78.3 (75.2−83.3)	77.04 (74.6−81)	78.7 (75.3−83.8)	0.353
Female sex, n (%)	66 (60.6)	12 (52.2)	54 (62.8)	0.355
Diabetes duration, years, median (IQR)	27.6 (17.3−35.7)	24.5 (16.6−35.2)	30.8 (18.5−38.3)	0.296
Diabetes type, n (%)				0.033
Type 1 diabetes	18 (16.5)	7 (30.4)	11 (12.8)	
Type 2 diabetes	89 (81.6)	15 (65.2)	74 (86.0)	
LADA	1 (0.9)	0 (0.0)	1 (1.2)	
Other diabetes	1 (0.9)	1 (4.3)	0 (0.0)	
Diabetic kidney disease, n (%)	67 (61.5)	13 (56.5)	54 (62.8)	0.583
Peripheral neuropathy, n (%)	58 (53.2)	12 (52.2)	46 (53.5)	0.911
Diabetic retinopathy, n (%)	43 (39.4)	11 (47.8)	32 (37.2)	0.355
Chronic ischemic cerebrovascular disease, n (%)	53 (48.6)	10 (43.5)	43 (50.0)	0.578
Coronary artery disease, n (%)	32 (29.4)	8 (34.8)	24 (27.9)	0.520
Peripheral arterial disease, n (%)	28 (25.7)	6 (26.1)	22 (25.6)	0.961
Hypertension, n (%)	101 (92.7)	22 (95.6)	79 (91.9)	1.000
Heart failure, n (%)	33 (30.3)	5 (21.7)	28 (32.6)	0.445
Dyslipidemia, n (%)	104 (95.4)	20 (87.0)	84 (97.7)	0.062
Atrial fibrillation, n (%)	22 (20.2)	4 (17.4)	18 (20.9)	1.000
MASLD, n (%)	63 (58.3)	9 (39.1)	54 (62.8)	0.055
Obstructive sleep apnea, n (%)	16 (14.7)	2 (8.7)	14 (16.3)	0.515
Obesity (BMI ≥ 30 kg/m^2^), n (%)	41 (37.6)	2 (8.7)	39 (45.3)	0.001
BMI, mean (SD), kg/m^2^	28.9 (5.8)	25.8 (5.0)	29.8 (5.7)	0.003
Metabolic syndrome, n (%)	92 (84.4)	14 (60.9)	78 (90.7)	0.001
HbA1c, mean (SD), %	8.1 (1.4)	7.7 (1.1)	8.3 (1.5)	0.071
Total daily insulin dose, IU, median (IQR)	39 (24−64.5)	31.5 (24−42)	40 (24−66)	0.230

Values are expressed as mean (SD), median (IQR), or n (%). The P value for diabetes type refers to the global comparison across categories. Abbreviations: BMI, body mass index; HbA1c, glycated hemoglobin; IQR, interquartile range; IU, international units; LADA, latent autoimmune diabetes in adults; MASLD, metabolic dysfunction-associated steatotic liver disease; SD, standard deviation.

**Fig. 1:**
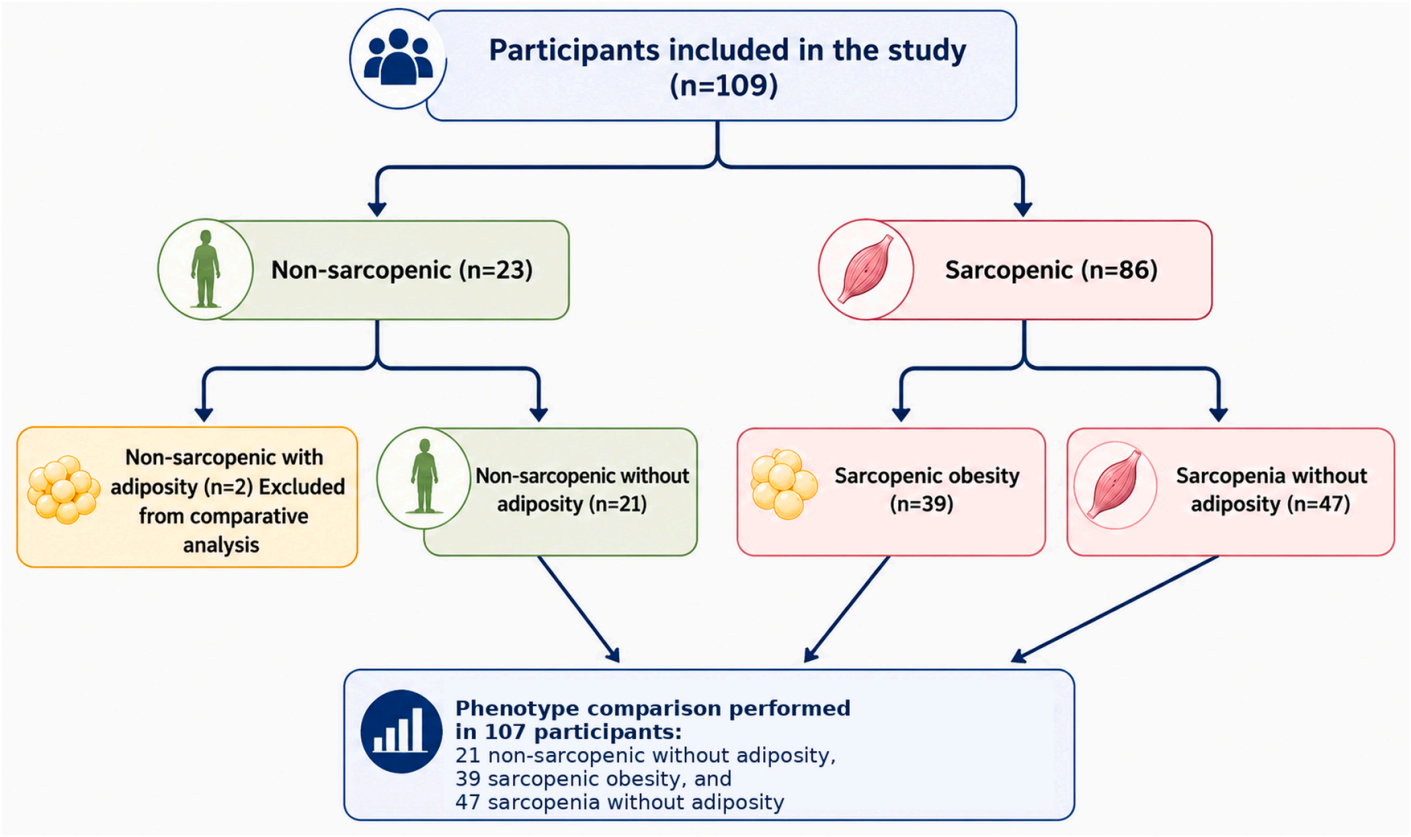
Study flowchart and phenotype allocation. Of 109 participants included in the study, 23 were classified as non-sarcopenic and 86 as sarcopenic. After exclusion of the 2 participants in the non-sarcopenic with adiposity subgroup, phenotype comparisons were performed in 107 participants: 21 non-sarcopenic without adiposity, 39 sarcopenic obesity, and 47 sarcopenia without adiposity.

After exclusion of the 2 participants with non-sarcopenic adiposity, 107 participants were included in phenotype comparisons: non-sarcopenic without adiposity (n = 21), sarcopenic obesity (n = 39), and sarcopenia without adiposity (n = 47) (Fig. 1). The sarcopenia without adiposity group was older (median 79.5 years [IQR, 76.5−84.8]) than the sarcopenic obesity (77.0 [73.0–79.6]) and non-sarcopenic without adiposity groups (77.7 [75.0–82.0]; P = 0.012). Diabetes type also differed (P = 0.013), with type 2 diabetes predominating in sarcopenic obesity (94.9%).

Sarcopenic obesity was characterized by greater central and total adiposity (waist circumference 115.4 cm, BMI 33.2 kg/m^2^, and fat mass 37.2%), higher triglycerides (146 mg/dL) and TyG index (9.4), universal metabolic syndrome, and greater total daily insulin dose (60 IU [IQR, 39.5−88]; global P ≤ 0.005 for each comparison). Glucagon-like peptide-1 receptor agonist use was also more frequent (35.9%; P = 0.001) (Table 2).

**Table 2:** Clinical, diabetes-related, and metabolic characteristics according to body composition-defined sarcopenia phenotype.

Variable	Non-sarcopenic without adiposity (n = 21)	Sarcopenic obesity (n = 39)	Sarcopenia without adiposity (n = 47)	P
Age, median (IQR), years	77.7 (75−82)	77 (73−79.6)	79.5 (76.5−84.8)	0.012
Diabetes type, n (%)				0.013
Type 1 diabetes	7 (35.0)	2 (5.1)	10 (21.3)	
Type 2 diabetes	13 (65.0)	37 (94.9)	37 (78.7)	
Diabetes duration, years, median (IQR)	32.5 (18.2−41.3)	23.1 (14.7−33.7)	28.5 (17.1−35.7)	0.383
HbA1c, mean (SD), %	7.6 (1.0)	8.3 (1.9)	8.3 (1.1)	0.209
CGM time in range, mean (SD), %	63.3 (19.3)	66.8 (25.4)	54.9 (14.6)	0.029
CGM time above range, mean (SD), %	33.8 (18.4)	30.5 (25.5)	41.6 (16.3)	0.053
CGM time below range, median (IQR), %	1 (1.0−5.0)	1 (0.0−4.0)	2 (0.0−6.0)	0.345
CGM coefficient of variation, mean (SD), %	32.7 (9.3)	31.4 (8.5)	32.8 (6.5)	0.749
GLP-1 RA use, n (%)	2 (9.5)	14 (35.9)	3 (6.4)	0.001
Total daily insulin dose, IU, median (IQR)	27.5 (23.6−39)	60 (39.5−88)	30 (21−48.5)	<0.001
Triglycerides, median (IQR), mg/dL	84 (49−227)	146 (79−542)	115 (56−504)	0.005
MetS, n (%)	12 (57.1)	39 (100.0)	39 (83.0)	0.005
TyG index, mean (SD)	8.6 (0.7)	9.4 (0.7)	9.1 (0.7)	0.005
BMI, median (IQR), kg/m^2^	24.8 (22.2−27.6)	33.2 (31.7−36.4)	26.0 (24.2−27.2)	0.005
Waist circumference, mean (SD), cm	94.5 (12.0)	115.4 (21.1)	97.3 (8.3)	0.001
Waist-to-height ratio	0.61	0.75	0.61	0.005
Fat mass, mean (SD), %	22.7 (5.4)	37.2 (7.8)	28.6 (6.2)	0.005
SMM/w, mean (SD)	0.31 (0.05)	0.25 (0.03)	0.28 (0.03)	0.001
Peripheral neuropathy, n (%)	11 (52.4)	15 (38.5)	31 (65.9)	0.039
u-ACR, median (IQR), mg/g	25 (11−205)	16 (5−120)	66 (19−224)	0.022
Chronic ischemic cerebrovascular disease, n (%)	8 (38.1)	14 (35.9)	29 (61.7)	0.036

Values are expressed as mean (SD), median (IQR), or n (%). Phenotypes were defined according to sarcopenia status and excess adiposity status. For phenotype comparisons, LADA was grouped with type 1 diabetes, and 1 participant with other diabetes was excluded from the diabetes type comparison; percentages for diabetes type are therefore based on 20, 39, and 47 participants, respectively. Abbreviations: BMI, body mass index; CGM, continuous glucose monitoring; GLP-1 RA, glucagon-like peptide-1 receptor agonist; HbA1c, glycated hemoglobin; IQR, interquartile range; IU, international units; MetS, metabolic syndrome; SMM/w, skeletal muscle mass adjusted by weight; TyG, triglyceride-glucose index; u-ACR, urinary albumin-to-creatinine ratio.

By contrast, sarcopenia without adiposity was characterized by more peripheral neuropathy (65.9%), higher urinary albumin-to-creatinine ratio (66 mg/g [IQR, 19−224]), and a higher prevalence of chronic ischemic cerebrovascular disease (61.7%; P ≤ 0.039 for each comparison). It also showed lower CGM time in range (54.9% [SD, 14.6]; P = 0.029) and numerically higher time above range (41.6% [SD, 16.3]; P = 0.053) despite HbA1c values similar to those in sarcopenic obesity (8.3% in both groups). Time below range and coefficient of variation did not differ significantly (Table 2).

In the exploratory adjusted model for poor CGM time-in-range control, HbA1c was the only variable significantly associated with the outcome (odds ratio [OR] 2.60, P = 0.002). Sarcopenia/adiposity phenotype was not significantly associated with poor time-in-range control overall (P = 0.100). In pairwise comparisons using sarcopenia without adiposity as the reference, sarcopenic obesity showed lower odds of poor time-in-range control (OR 0.26, P = 0.048), whereas non-sarcopenic without adiposity did not differ significantly (OR 0.31, P = 0.105).

## 4 Discussion

This short communication describes a clinically complex cohort of frail older adults with diabetes in whom sarcopenia was highly prevalent and metabolically heterogeneous. The main observation is not the prevalence estimate alone, but the separation of body composition-defined sarcopenia phenotypes into distinct diabetes-related, metabolic, and CGM-derived profiles.

The high sarcopenia prevalence should be interpreted in the context of an older, frail, multimorbid cohort with long-standing diabetes, and is higher than estimates reported in broader community-based samples [22]. The clinical relevance of sarcopenia and sarcopenic obesity is supported by meta-analytic evidence linking these conditions to adverse outcomes, although the present study was not designed to evaluate prognosis [23].

Sarcopenic obesity showed a cluster of metabolic abnormalities associated with insulin resistance, including increased central adiposity, triglycerides, TyG index, metabolic syndrome prevalence, and insulin requirements. This pattern supports the biological plausibility of an insulin resistance-related phenotype [7,8,13]. Nevertheless, insulin sensitivity was not directly measured, and the TyG index is partly determined by fasting glucose and triglyceride concentrations; these findings therefore represent indirect markers rather than direct evidence of insulin resistance.

The absence of overt adiposity did not identify a metabolically neutral phenotype. Sarcopenia without adiposity was characterized by greater peripheral neuropathy, albuminuria, a higher prevalence of chronic ischemic cerebrovascular disease, and lower CGM time in range despite HbA1c values similar to those in sarcopenic obesity [24,25]. This finding supports the value of CGM in identifying glycemic heterogeneity that may not be captured by HbA1c alone [26].

The exploratory adjusted model indicated that HbA1c, rather than phenotype, was the only independent correlate of poor time-in-range control. This may indicate that glycemic burden, rather than body composition phenotype alone, was the principal correlate of CGM impairment in this sample. The pairwise signal suggesting lower odds of poor time-in-range control in sarcopenic obesity than in sarcopenia without adiposity remains hypothesis-generating because the overall phenotype effect was not significant and the sample was limited.

From a clinical perspective, these phenotypes may have different management implications. In sarcopenic obesity, interventions addressing excess adiposity and hyperglycemia should preserve muscle mass and function, with resistance exercise and individualized nutritional support as relevant components [7,8]. In sarcopenia without adiposity, weight-loss strategies should be individualized rather than routinely applied; emphasis may instead be placed on maintaining physical function, individualized glycemic targets, CGM review, and surveillance of diabetes complications [[26], [27], [28]]. Exercise-oriented guidance and observational evidence support preserving physical function across phenotypes [29,30]. These implications are hypothesis-generating and do not constitute validated phenotype-specific treatment recommendations.

Several limitations must be emphasized. The study was cross-sectional and single-center, subgroup sizes were modest, and the non-sarcopenic with adiposity group was too small for meaningful comparison. Analyses were primarily descriptive, and the adjusted model was exploratory. Frailty was defined pragmatically using a combined approach within a real-world clinical program, and body composition was assessed by bioelectrical impedance rather than dual-energy X-ray absorptiometry. Insulin resistance was not directly measured and longitudinal clinical outcomes were not assessed; consequently, the prognostic and therapeutic implications of the phenotypes remain uncertain. Residual confounding by diabetes type, duration, and treatment cannot be excluded.

Despite these limitations, the study integrates body composition, diabetes-related complications, insulin resistance-related markers, and CGM in an understudied clinical population. In this small real-world cohort, sarcopenic obesity showed an insulin resistance-related cluster, whereas sarcopenia without adiposity showed greater complication burden and lower time in range. Larger longitudinal studies should determine whether these phenotypes differ in clinical outcomes and whether phenotype-aware management improves care.

## CRediT authorship contribution statement

Ernesto Guevara: Conceptualization, Methodology, Investigation, Data curation, Writing - original draft, Writing - review & editing.

Andreu Simó-Servat: Data interpretation, Writing - review & editing.

Verónica Perea: Data interpretation, Formal analysis, Writing - review & editing.

Carmen Quirós: Data interpretation, Writing - review & editing.

Carlos Puig-Jové: Data interpretation, Writing - review & editing.

Francesc Formiga: Conceptualization, Methodology, Supervision, Validation, Writing - review & editing.

María-José Barahona: Conceptualization, Methodology, Formal analysis, Supervision, Validation, Writing - review & editing.

All authors read and approved the final manuscript.

## Declaration of Generative AI and AI-assisted technologies in the writing process

During the preparation of this work, the authors used ChatGPT (OpenAI) to support language editing. After using this tool, the authors critically reviewed and edited the content as needed and take full responsibility for the content of the article.

## Funding

This research received no specific grant from any funding agency in the public, commercial, or not-for-profit sectors.

## Data availability

The datasets generated and/or analyzed during the current study are not publicly available due to institutional data protection regulations and the potentially identifiable nature of clinical data, but are available from the corresponding author on reasonable request and subject to applicable institutional and ethical requirements.

The data that has been used is confidential.

Data will be made available on request.

## Declaration of competing interest

The authors declare that they have no known competing financial interests or personal relationships that could have appeared to influence the work reported in this paper.
